# The Influence of Cheeks and Tongue and Lips on Gums and Jaws

**Published:** 1878-07

**Authors:** C. Sauer


### ARTICLE IV.

### *The Influence of the Cheeks and Tongue, and the Lips and Tongue on the Gums and Jaws.*

BY C. SAUER.

[A TRANSLATION.]

The pain following extraction of the teeth, upon which I published a paper in the year 1874, in the *Deutche Viertel-jahrsshrift fur Zahnheilkunde*, has given me occasion to make further observation of known physiological processes, and the influence exerted by the lips and tongue, and cheeks and tongue, upon the gums and alveolar ridge. I take occasion to present the following short consideration of pain after extraction.

Pain immediately following extraction of teeth deserves our special notice on account of its severity; it is sometimes much more severe than the toothache was before extraction.

For us dentists it is all the more important for the reason that patients believe that we are to blame for it. We must therefore give it our closest attention. Heretofore we have been quite powerless against it. We looked only to the irritation of the broken nerve in the maxilla or an induced ostitis as the cause. The pain might arise from these causes but in my cases ostitis only has occasionally been present. I have always found the seat of pain in the border of the gums corresponding to the edges of the alveoli. The gums always rise above the bony walls of the alveoli, and in these cases the pain arises as soon as the gums are pressed against these margins. There is no pain in the alveoli. As in these cases there is little or no blood clot, I unite the absence of this with the painful gum at the alveolar margin, for in such a case I am of the opinion, that there being no counteracting pressure from within the alveolus against the tongue and cheeks, or the tongue and lips, these press the gums in over the sharp alveolar borders and thus give rise to inflammation.

A few days pass during which patients suffer without seeking aid, and then it is found that the margins of the gums are so bent over the alveolar processes from either side that they almost touch. Occasionally the pain comes on soon after extraction; this seems to arise from the inflammation of the gum about the diseased tooth before extraction; this is seen especially in cases of pericementitis. In such cases it is easy to understand how the inflamed gum may quickly become painful under the direct action of an irritant. We may also understand how the gums alone may become painful without the necessity of attributing the cause to a broken nerve in the many cases of exceeding painfulness of the gums that may be induced about a loose piece of a root. In such cases we certainly cannot attribute it to an injured nerve.

The treatment of pain after extraction should be upon the above theory. Remove the hurtful influence of the tongue, lips and cheeks. In the milder cases this may be done by filling the socket of the tooth with loose pledgets of cotton. These have been made use of heretofore but they were saturated with iodine, laudanum, etc., and it was supposed that the benefits were derived from these medicaments. If the pain be great, over-capping the gums so as to ward off the lips and tongue will be beneficial. Together with plugging the alveolus, a cut in the gums, corresponding to the border of the process will be found useful—this seems of value when ostitis is also present; by this course the gums are scarified. I also use moist warmth as an aid in the treatment.

The unpleasant influence of the movable parts of the mouth on the gum, in the manner above described, is best observed after the extraction of the lower wisdom teeth. This may also be shown in many cases besides at the margins of the gums on the borders of the alveolar processes—sharp cusps of teeth under the gums—breaks in the alveoli, and bony borders after resection show it very often, as do foreign bodies in the gums, as fish-bones, broken pieces of tooth-picks, etc. Calculus may also be an occasional cause.

Cases of pain following the extraction of the wisdom teeth have, on account of the great movableness of the neighboring tissues, directed me to the observation of this class of cases, and aroused me to prosecute their study. It was through this that I came to connect the cause of pain after extraction, and the cause of the pain which occurs so frequently in the cutting of the wisdom teeth. It is my opinion that the wisdom tooth does not cause pain so much by pressure upon the gums in its effort to pass through, as heretofore supposed, as by the dragging of the gums across the cusps of its grinding surface. In this view of the case a cross cut down to the grinding surface, or a removal of the gums over the tooth, will be of advantage. By this means the neighboring soft parts will be prevented from dragging and pressing the gums against the cusps of the tooth. Of course this does not include those cases in which trouble arises from pressure against the next tooth in front.

I also hold that in the cutting of the milk teeth severe inflammation of the gums may be caused in the same manner as in the cutting of the wisdom teeth.

The coming of the permanent teeth sometimes suffers irregularity which I ascribe, at least in part, to the influence of the lips, cheeks and tongue. In the shedding of the milk teeth it is the rule that they fall out, or are removed as soon as the coming permanent tooth is level with the gums. The front teeth show themselves, as a rule, just behind the milk teeth, the back teeth immediately beneath them. The irregularity of which I have spoken occurs only in case the milk teeth fall out before the permanent teeth have reached the height of the gums. This premature shedding I ascribe to fungous growths of the gums, which I believe to be caused by pressure against the edges of the absorbed surface of the milk teeth, which in these cases grow in under the milk tooth and loosen it prematurely; finally the fungoid growth entirely breaks up the slight hold of the milk tooth and causes it to fall out. At this time the permanent tooth has reached the height of the bony border of the alveolus beneath the gums. After the loss of the milk tooth the gums close quickly over the coming permanent tooth; within two or three days only a slight slit is seen over the coming permanent tooth, which is already beginning to unite, through which a sharp instrument can scarcely be passed without injury. The lips and tongue have here induced a condition which retards the cutting of the permanent teeth. In the shedding of the milk molars we find in such cases the gums stretching like a band from the prominences of the alveolar border on either side across between the adjoining teeth.

If in such premature shedding of the teeth, the neighboring teeth, on one or both sides, have been exchanged, then it may happen that these take too much room and the new tooth which comes through later, not finding sufficient room must take a wrong position. Heretofore we have done nothing to correct this, probably because it was supposed that the tooth was not sufficiently developed to come forward ; this however is not the case. In such cases we often see the corresponding tooth on the opposite side, which has not been retarded by the gums, taking its place in the arch. As the development of the corresponding tooth is usually equal, we must suppose that the retarded tooth is equally well developed.

The treatment is very simple when the cause is understood. I hold the borders of the gums open by a tent of cotton ; occasionally I make room by a cut and prevent its healing by the use of tents. If the coming tooth be overgrown by a band-like gum, I cut this through and cauterize it with nitrate of silver and also introduce tents ; the wound must be kept open for some time. Since adopting this treatment I have been surprised to see such teeth coming rapidly forward.

We occasionally see a milk tooth still remaining in the row of permanent teeth in persons who have passed the time of shedding. The corresponning permanent tooth has, from some cause—as its position—been held back in the maxilla. Hitherto, if such a milk tooth was removed for any reason —as on account of pericementitis—we gave ourselves no trouble about the retarded permanent tooth, especially if there was sufficient room in the arch for it to come forward. After the observations I have made as to the cutting of the permanent teeth at the time of shedding, I notice closely the roots, and also the alveolus of such teeth, and if I find the least absorption of the root, or can feel in the depths of the alveolus the crown of the delayed permanent tooth, I hold the alveolus open by tents.

There have been cases in which the jaw had remained toothless for years, and in which at last a retained permanent tooth made its appearance. I have observed two such cases. In these I saw the belated permanent teeth come through the gums in persons 35 and 40 years old, and who had worn artificial teeth on toothless and rootless gums for 5 and 10 years. These teeth were, in the one case, a cuspid, in the other a right superior bicuspid, each of which came through the gums and assumed its proper position in the mouth. I ascribe the final eruption of these belated teeth to an irritation of the gums which had extended to the peridental membrane. The bone was brought to a condition favorable to the coming forward of the retained teeth, perhaps through growth of its spongy parts. In both of these cases the irritation seemed to have been caused by the base of the artificial teeth, partly through its movements in chewing and partly through the continued influence of the tongue, lips and cheeks.

The disappearance of the alveoli, and the closure of the gums over them may also be ascribed in part to the influence of the cheeks and tongue. The alveolar borders are in this wise pressed together and the gums pressed over them until they come together and unite. After the disappearance of the alveoli the alveolar ridge takes—under the influence of the tongue, lips and cheeks—a V or roof shape. In toothless mouths this is especially noticeable in the lower jaw. Here the tongue and cheeks are continually lying against each other. In the upper jaw the form is correspondingly more rounded. The roof form (sharp ridge) in both upper and lower jaw is seen more in the position of the back teeth, while it is more rounded in the position of the cuspids and incisor teeth. The cause of this is to be found in the position of the tongue, which lies more in the deeper parts of the mouth, and is brought less forcibly against the lips.

By the unequal pressure of a poorly fitting denture on the lower jaw, the union of the cheeks and tongue with the jaw, at a point corresponding to the back teeth, can be considerably raised up ; it may be owing to the inclination of the patient to avoid this unnatural pressure by means of his cheeks and tongue, *i. e.*, drawing them backward. The cheek and tongue then form a fold or flap that covers the jaw. A well fitting plate, worn patiently, can force this covering back into place. The plate must be worn from a month to a year, or even, it may be, for a longer time. By the help of the plate the soft parts give away, allowing it to rest upon the unusually shrunken jaw. This point of union, then, is found to be lower down, or, in its normal place. The base of the denture must, of course, be now renewed. According to this we must admit that the use of a well fitting set of teeth, can both hinder an immoderate shrinking of the jaw, and oppose a too great influence of the cheek and tongue.

The object of this paper is to point out how manifold changes may be brought about by the influence of cheeks and tongue and lips and tongue in certain conditions of the gums and jaws, and to recommend further observation and reporting of cases of this nature.—*Missouri Dental Jour.*

